# Sleep Pattern Changes in Patients Who Are Scheduled for Cardiac Surgery

**DOI:** 10.7759/cureus.48543

**Published:** 2023-11-08

**Authors:** Syed Shabbir Ahmed, Mohammad Hamid, Muhammad S Yousuf, Saqiba Tahir

**Affiliations:** 1 Anaesthesiology, Aga Khan University Hospital, Karachi, PAK

**Keywords:** sleep disturbance, surgical patients, cardiac surgery, sleep pattern, sleep deprivation

## Abstract

Introduction

Surgery patients frequently experience sleep deprivation, which is regarded as a stress factor during the perioperative period and can cause physical discomfort, exhaustion, and even postoperative pain. There is a dearth of information on preoperative sleep habits and the consequences that may result. There are both subjective and objective ways to rate the quality of your sleep. We chose the Pittsburgh Sleep Quality Index (PSQI), which employs a questionnaire to provide crucial information on issues like sleep length, efficiency, and interruption. Lower sleep quality is correlated with higher PSQI scores.

Study objective

Our study aimed to assess the changes in the sleep pattern of cardiac disease patients before cardiac surgery and compare these changes with baseline sleep patterns.

Methods

This prospective survey was carried out after ethical review committee approval at the Department of Anesthesia, Aga Khan University Hospital. Consent was obtained from all patients undergoing cardiac surgery. Strict inclusion and exclusion criteria were followed.

All patients aged 25 to 65 who came from home for elective cardiac surgery under general anesthesia were included. At the same time, patient demographics were noted. Additionally, a printed PSQI questionnaire was distributed to each participant. The native Urdu language was also translated into this questionnaire. The patient was given an explanation of this form by a medical professional. This questionnaire was filled out by the patients on the surgical floor or preoperative area before premedication. The PSQI questionnaire was used to assess baseline sleeping patterns, and then the same questions were asked about the period between decisions for the date of surgery and the time of admission for surgery.

Results

A total of 83 patients participated in the study. The mean age of the patient was 57 (*±*13.0), out of which 67 (80.7%) were males and 16 (19.3%) were females. The most common surgeries were coronary artery bypass (CABG) surgery 63 (77.8%), followed by valve replacement nine (11.1%).

The overall mean PSQI score was higher (5.27) once the surgery date was decided as compared to the baseline (4.84), but it did not reach the statistically significant level (p-value 0.411). Sleep latency (time to fall asleep while in bed) was the only variable statistically significant between baseline (26.1 (±35.0) and after the surgery date has been finalized (36.1 (±46.6)).

No significant differences were found in other variables like sleep quality (feeling of being well-rested), sleep duration (hours of actual sleep), sleep efficiency (sleep efficiency is the ratio of the amount of total time asleep versus the total time in bed), and sleep disturbance (problem initiating and maintaining sleep). Total bedtime was also reduced at night before surgery but did not achieve a significant level. The logistic regression model demonstrated that age, gender, and type of surgery did not influence sleep quality.

Conclusion

In the present study, lower sleep quality was observed before cardiac surgery, but it did not reach a statistically significant level when compared with baseline. Sleep latency (time to fall asleep while in bed) was significantly prolonged compared to baseline. We could not find any association between quality of sleep and variables like age, gender, and type of surgery.

## Introduction

Sleep deprivation is considered a common stress factor among surgical patients in the lead-up to surgery and can cause physical discomfort, exhaustion, and even postoperative pain. On preoperative sleep patterns and their corresponding adverse effects, there is a dearth of literature. Hospitalized patients experience sleep disturbances more frequently than patients who are admitted on the day of surgery. which may be connected to worry, discomfort, the complexity of the surgery, and other environmental variables [1-6].

Preoperative sleep needs should be met in order to increase patient satisfaction and quality of life. It is critical to understand the patient's baseline sleep pattern and sleep quality while assessing preoperative sleep. There are both subjective and objective ways to rate the quality of sleep. Actigraphy and polysomnography are frequently employed for objective sleep monitoring. While a questionnaire is frequently used to evaluate patients’ subjective sleeping habits. The Pittsburgh Sleep Quality Index (PSQI) employs a questionnaire to provide information on important issues, including sleep length, efficiency, and disruption. Sleep quality is inversely correlated with higher PSQI scores. The many aspects of sleep quality include sleep initiation, maintenance, depth, quantity, dreams, state following sleep, and impact on everyday living [5].

Sleep disturbance is probably common once the patient is admitted for surgery. However, there is little research on the subject and no management recommendations. The impact of preoperative sleep disruptions on various postoperative components has only been briefly discussed in the research. The degree of postoperative pain appears to be exacerbated more by interrupted sleep the night before surgery than by other sleep-related variables. It could also have an impact on postoperative quality of life. Even when they slept at home, Leung et al. discovered that patients who experienced surgical delirium had higher preoperative sleep problems [6].

High PSQI ratings for three consecutive days before surgery and even higher values in the postoperative phase were noted in another study on patients undergoing heart surgery [7]. It is necessary to develop strategies to address this predicted issue by providing these patients with preoperative care while also employing sedatives or other drugs.

The objective of our study is to assess the changes in sleep patterns of cardiac disease patients before cardiac surgery (once the surgery date is decided) and compare these changes with baseline sleep pattern.

## Materials and methods

This prospective survey was carried out after ethical review committee approval at the Department of Anaesthesia, Aga Khan University Hospital. Consent was obtained from 83 patients undergoing cardiac surgery, included on the basis of a previous study that revealed a pattern of relatively low nocturnal sleep efficiency (79.3%) before surgery, with a 9% margin of error and a 95% confidence interval [7]. Strict inclusion and exclusion criteria were followed. All elective cardiac surgery patients between the ages of 25 and 65 years were considered, while patients who were in severe pain, patients on sleeping pills, psychiatric patients, sleep apnea patients, patients with sleep disorders, and patients who were unable to complete the questionnaire were excluded from this study. At the same time, patient demographics were noted.

Additionally, a written PSQI questionnaire was given to each participant to complete. The local Urdu language was also translated into this questionnaire. The patient was explained this form by a medical professional. This questionnaire was filled out by the patients on the surgical floor or preoperative area before premedication. The PSQI questionnaire was used to assess baseline sleeping patterns, and then the same questions were asked about the period between decisions for the date of surgery and the time of admission for surgery.

We conducted the statistical analyses using RStudio (version 4.1.2; Boston, USA). To assess normality, we employed the Shapiro-Wilks test. Qualitative variables, such as gender and surgery type, were evaluated using frequency and percentage, while quantitative data, including age, total time in bed (in hours), total sleep time (in hours), sleep latency (in minutes), and more, were summarized using mean and standard deviation. To determine the significance of changes in patients' sleep patterns before and after surgery, we used paired t-tests for quantitative variables and Chi-square or Fisher exact tests for qualitative variables. In cardiac surgery patients, both univariable and multivariable binary logistic regression models were employed to identify factors associated with poor sleep, defined as a PSQI score of 5 or higher. We considered p-values of ≤0.05 as statistically significant.

## Results

A total of 83 patients participated in the study. The mean age of the patients was 57 (13.0), out of which 67 (80.7%) were males and 16 (19.3%) were females (Table 1). The most common surgery was coronary artery bypass (CABG) surgery 63 (77.8%), followed by valve replacement nine (11.1%).

**Table 1:** Demographic and surgery statistics (n = 83)

Age	
Mean (SD)	57.0 (13.0)
Median [Min, Max]	58.5 [18.0, 77.0]
Gender	
Male	67 (80.7%)
Female	16 (19.3%)
Surgery	
CABG	63 (77.8%)
Other	18 (22.2%)

Fifteen (12.6%) patients had no partner or roommate; 33 (27.39%) patients had partners sleeping on the same bed, while the rest of the patients had partners either sleeping in an adjacent room or in the same room in a separate bed.

**Figure 1:**
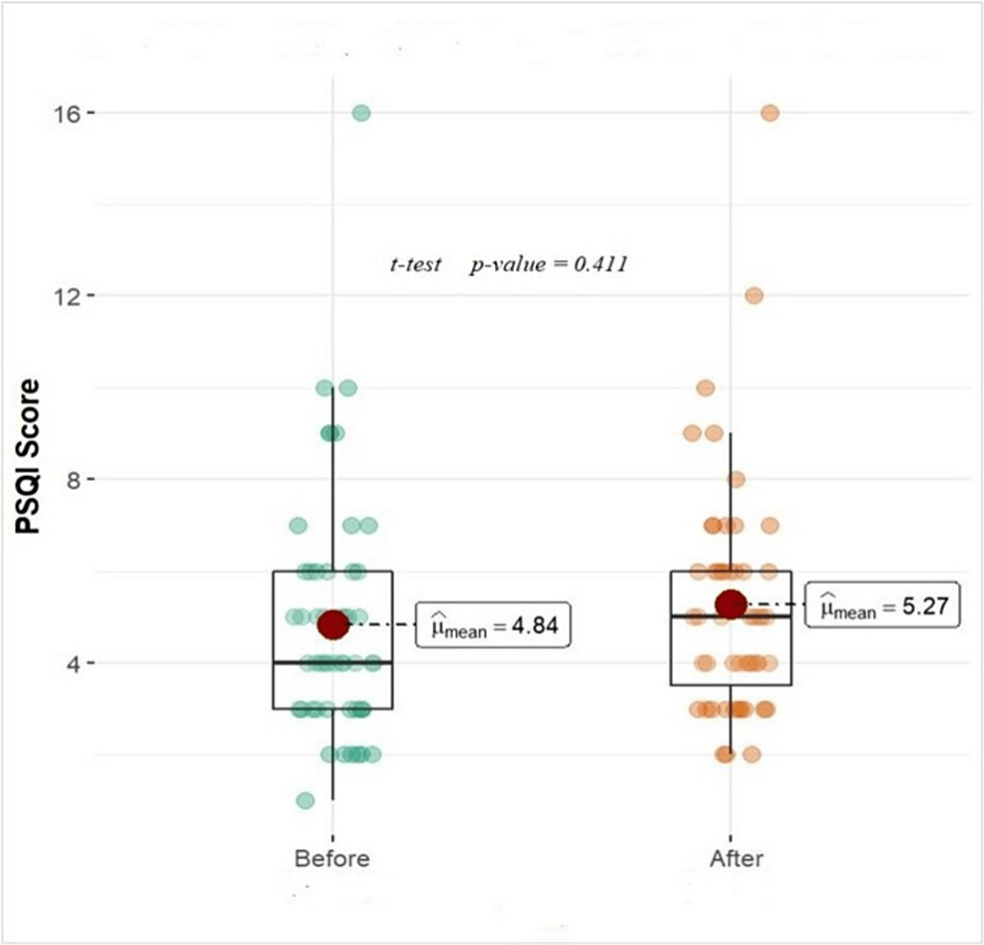
Comparison of PSQI between baseline sleep patterns before surgical date decision and after surgery date has been decided

Overall mean PSQI scores (Figure 1) were higher (5.27) once the surgery date was decided when compared with baseline (4.84), but they did not reach a statistically significant level (p-value 0.411).

Sleep latency was the only variable that was statistically significant between baseline 26.1 (35.0) and after the surgical date decided 36.1 (46.6).

No significant differences were found in other variables like sleep quality, sleep duration, sleep efficiency, and sleep disturbance. Total bedtime was also reduced at night before surgery but did not achieve a significant level (Table 2).

**Table 2:** Assessment of sleep patterns for cardiac surgery patients (n = 83)

Variables	Before	After	P-value
Total time in bed (hours)			
Mean (SD)	7.51 (1.41)	7.32 (1.62)	0.061^*^
Total sleep time (hours)			
Mean (SD)	7.23 (1.54)	7.02 (1.63)	0.102^*^
Sleep latency (minutes)			
Mean (SD)	26.1 (35.0)	36.1 (46.6)	<0.001^*^
Subjective sleep quality [C1]			
Very good	21 (25.6%)	16 (26.7%)	0.991^⸸^
Fairly good	48 (58.5%)	35 (58.3%)	
Fairly bad	7 (8.54%)	4 (6.67%)	
Very bad	6 (7.32%)	5 (8.33%)	
Sleep latency [C2]			
0	22 (26.5%)	16 (27.1%)	0.990^⸸^
1 to 2	35 (42.2%)	26 (44.1%)	
3 to 4	18 (21.7%)	12 (20.3%)	
5 to 6	8 (9.64%)	5 (8.47%)	
Sleep duration [C3]			
> 7 hours	39 (50.6%)	34 (54.0%)	0.933^⸸^
6-7 hours	17 (22.1%)	14 (22.2%)	
5-6 hours	11 (14.3%)	9 (14.3%)	
< 5 hours	10 (13.0%)	6 (9.52%)	
Sleep efficiency [C4]			
> 85%	62 (75.6%)	55 (82.1%)	0.7614^⸸^
75-84%	5 (6.10%)	4 (5.97%)	
65-74%	5 (6.10%)	3 (4.48%)	
< 65%	10 (12.2%)	5 (7.46%)	
Sleep disturbance [C5]			
1 to 9	52 (62.7%)	46 (73.0%)	0.3862^⸸^
10 to 18	28 (33.7%)	15 (23.8%)	
19 to 27	3 (3.61%)	2 (3.17%)	
Use of sleep medication [C6]			
Not during past month	65 (78.3%)	51 (83.6%)	0.810^⸸^
Less than once a week	8 (9.64%)	5 (8.20%)	
Once or twice a week	4 (4.82%)	1 (1.64%)	
Three or more times a week	6 (7.23%)	4 (6.56%)	
Daytime dysfunction [C7]			
0	45 (54.2%)	41 (67.2%)	0.2876^⸸^
1 to 2	27 (32.5%)	17 (27.9%)	
3 to 4	9 (10.8%)	3 (4.92%)	
5 to 6	2 (2.41%)	0 (0.00%)	
Note; * Paired t-test; ⸸ Chi-Square or Fisher Exact test			

The logistic regression model demonstrated that age, gender, and type of surgery had no influence on the quality of sleep (Table 3).

**Table 3:** Predictors of poor sleep (PSQI ≥5) by logistic regression analysis

Predictor	Crude OR (95%CI)	P-value	Adj. OR (95%CI)	P-value
Age	0.97 (0.92,1.02)	0.23	0.98 (0.92,1.03)	0.394
Sex (Female; Male)	0.63 (0.16,2.5)	0.513	0.46 (0.07,2.92)	0.411
Surgery (CABG; Other)	1.22 (0.31,4.89)	0.777	2.6 (0.39,17.35)	0.323
Sleep pattern (After; Before)	1.73 (0.93,3.22)	0.073	1.74 (0.93,3.26)	0.082

## Discussion

Sleep is a regular, naturally occurring state of relaxation marked by lowered metabolism, partial or total loss of awareness, and diminished or absent sensory and voluntary muscle activity. Sleep also helps to save energy. Age, gender, and the foods we eat, as well as our physical and mental health, all have an impact on how well we sleep. Sleep disruption has an impact on daily activities, health, and overall quality of life [4,8-9].

Sleeping well is important for survival and makes it easier to recover from surgery since it renews both physical and mental energy. Health is significantly affected by sleep disorders, as they impair immunological function and raise mortality and cardiovascular disease risks. According to reports, between 47% and 68% of coronary artery bypass graft (CABG) patients experience sleep difficulties. These consist of lack of sleep, tiredness throughout the day, poor sleep quality, and insomnia [10-11]. Literature on preoperative sleep patterns in surgery patients is scarce.

This research is to examine the preoperative sleep habits and sleep quality of patients undergoing cardiac surgery in our population. To evaluate the quality of sleep and disturbances throughout a month, we utilized the Pittsburgh Sleep Quality Index (PSQI), a self-reported questionnaire that is often used. Sleep quality is inversely correlated with higher PSQI scores. A PSQI of 5 or more is regarded as having poor sleep quality [9-10]. We compared last month's sleep activity as a baseline before and after the surgery date was decided. Overall mean PSQI scores (Figure 1) were higher (5.27 after surgery date) when compared with baseline, but they did not reach a statistically significant level. Sleep latency, which was statistically significant between baseline and after the surgery date, has been finalized. Sleep latency can be affected in heart surgery patients by a variety of things, including pain, anxiety, and drug use, all of which are related to preoperative as well as routine postoperative issues. According to studies, patients who undergo heart surgery typically have higher sleep latency postoperatively than healthy people. This might be the result of things like pain from the surgical incision, discomfort from the healing process, and procedure-related anxiety. Healthcare professionals may suggest the use of pain management techniques, such as non-opioid painkillers, to alleviate discomfort and encourage sleep in order to address sleep delays in heart surgery patients. Additionally, it may be advised to use relaxation techniques like deep breathing, guided meditation, or progressive muscular relaxation [12]. All of these were explained well for the postoperative phase, and there was no clear data available for the preoperative phase, so this study showed significance in sleep latency before cardiac surgery. We couldn’t pinpoint the factors that caused these problems in the preoperative phase.

When compared to the baseline, which was evaluated six weeks earlier, Boaz and colleagues also showed that preoperative sleep is primarily affected at night before surgery and found that night before surgery sleep quality was noticeably different than the baseline levels. Gynecology patients' preoperative sleep habits were examined at home six weeks, two days, and the night before operation [13]. Bakry et al. found that immediately after having cardiac surgery, the quality of sleep was affected. Most patients who underwent open heart surgery experienced more sleep disruption, less restorative sleep, and greater daytime drowsiness and fatigue. Anxiety, depression, and increased weariness behavior with a tendency towards immobility following surgery were all linked to poor sleep quality after cardiac surgery. Fortunately, within one month, these alterations had returned to their preoperative levels, although they can take 6 to 12 months after surgery [14]. Although there is a lack of data on preoperative sleep assessment in cardiac surgery. The present study also showed lower quality of sleep before cardiac surgery but did not reach a statistically significant level.

The major causes of sleep disruption in cardiac surgery patients are surgical fear and anxiety. It begins when the patient is informed that surgery is necessary and increasingly worsens during the hospitalization process. Each patient's level of dread and anxiety varies depending on several variables, including their sensitivity, age, gender, past surgical experience, level of education, intended operation kind and extent, current health state, and socioeconomic situation [12].

The best way to assess the quality of sleep over the course of a month is probably through sleep efficiency. The sensitivity of measuring sleep over a month is presumably lower. Wright and colleagues also claimed that over 85% of sleep was efficient; however, this was not proven to be significant in this investigation. They focused on the relationship between sleep quality the night before surgery and postoperative pain management and discovered that patients with the lowest sleep quality had pain levels that were 59% greater than those with the best sleep quality, and that difference persisted for a week. The cause may be that we did not account for nighttime awakenings or sleep interruptions, which similarly shorten overall nighttime sleep duration. Given that this was a subjective assessment and that patients had trouble recalling the overall amount of time they had been interrupted. It's probably preferable to keep a sleep record for the entire month before answering this questionnaire [9]. Considerably, more than one sleep disruption will be considered a positive reaction, so it is probably inappropriate to compare last night's activity with that of the whole month. Therefore, in this study, we compared sleep disturbances in the last month before and after deciding of surgery but didn’t find any significance. Although there are no recommendations for addressing this problem, sleep disruption is frequently found before surgery. Despite the fact that the patient is sleeping in a familiar setting, the current investigation was unable to prove the existence of poor sleep quality before heart surgery.

Additionally, there are some limitations to this study. We were unable to get data on the psychological health and preoperative sleep quality of all patients. The nature of the hospital setting was to blame for this research design issue. Therefore, we are unable to draw the conclusion that heart surgery can affect sleep disruption. The choice of possible risk factors for logistic regression analysis is yet another drawback. Future research is required to increase our understanding of how much each risk factor contributes to the start of disturbed sleep quality. Additional research is required to examine the relationship between poor sleep quality and surgical outcomes, including satisfaction and pain management, as well as the necessity for intraoperative anesthesia.

## Conclusions

In the present study, lower quality of sleep was observed before cardiac surgery once the surgery date was decided, but it did not reach a statistically significant level when compared with baseline as the PSQI score did not reach the significance level. Sleep latency (time to fall asleep while on the bed) was significantly prolonged when compared with baseline. We were unable to find any association between quality of sleep and variables like age, gender, and type of surgery.
